# Conquering camouflage: a collaborative feature refinement detector for near-color backgrounds and small-size challenges

**DOI:** 10.3389/fpls.2026.1882013

**Published:** 2026-08-18

**Authors:** Shilin Li, Sheng Gao, Lili Sun, Nan Yang, Shujuan Zhang, Fuzhong Li

**Affiliations:** 1 Faculty of Software Technologies, Shanxi Agricultural University, Jinzhong, China; 2 College of Agricultural Engineering, Shanxi Agricultural University, Jinzhong, China

**Keywords:** collaborative feature refinement, lightweight algorithm, near-color recognition, selective picking, small object detection

## Abstract

Field detection of flat jujubes faces three coupled challenges: extreme color similarity between fruit and leaves, small fruit size, and the requirement of lightweight deployment on edge devices. Existing methods either address one of these problems in isolation or simply stack existing techniques without a principled design for the triple conflict. This paper proposes Collaborative Feature Refinement Detector (CFRDet), which synergistically integrates four modules to resolve the above conflicts. Mish activation preserves weak gradients, preventing small target features from vanishing in lightweight networks. The parameter-free Area-Attention Similarity-Aware Module (ASAM) suppresses near-color background interference without increasing parameters. A dedicated small target detection layer recovers spatial details lost during downsampling. Mobile Inverted Residual Bottleneck (MIRB) detection heads process the high-resolution feature map with nearly linear complexity, offsetting the computational cost of the small target layer. Together, they form a collaborative chain of “feature preservation, background suppression, detail recovery, low-cost computation”. Experiments on our self-built flat jujube dataset showed that CFRDet improved mAP and F1 by 1.7 and 3 percentage points respectively over a strong baseline, reduced FLOPs to 5.6 G, and compressed the parameters and model size to 2.3 MB and 5.1 MB. Comprehensive ablation studies verify the necessity of each module and their synergistic gains. In comparisons with mainstream models, it surpassed small-sized models in accuracy while retaining lower complexity than nano-sized networks, and its generalization was further confirmed on a sweet persimmon dataset. The model’s dual-attribute of precision and lightness underpins intelligent automation in field flat jujube harvesting.

## 1 Introduction

Target detection of fruits in field environments constitutes a fundamental and critical prerequisite for enhancing the efficiency of automated harvesting. However, with the growing market demand, automated harvesting poses two major challenges: (1) low detection accuracy for small-sized fruits detection. In complex agricultural environments, fruit images often exhibit multi-scale objects, where those in the background appear large and distinct, while those in the foreground are small and densely clustered. In these scenarios, the scarcity of effective pixels combined with the difficulty in leveraging contextual information leads to feature loss or submersion in small fruit targets, substantially compromising the performance of feature extraction. (2) difficulty in accurate fruit recognition against near-color backgrounds. The coloration of fruits at different maturity stages (such as chlorophyll-green and yellowish-green) closely resembles that of the background elements (leaves, canopy). This subtlety in feature differences hinders the model from learning discriminative fine-grained representations. Therefore, addressing the challenges of accurately detecting fruits against near-color backgrounds and improving the recognition precision for small target fruits is of significant research importance for enhancing automated harvesting efficiency and advancing smart agriculture.

Small object detection problem. With their area below 32×32 pixels in some dataset images, flat jujubes are categorized as small objects, meeting the COCO dataset criterion used in object detection. The Small Object Detection (SOD) task (Hua and Chen, 2025; Li et al., 2025) faces two main challenges: (1) Feature loss is highly likely to occur during multiple down-sampling processes. Accordingly, Hou et al. (2025) developed a lightweight small-object detection model based on a multi-branch feature fusion architecture. (2) Small objects have low contrast against complex backgrounds, posing a risk of feature submergence. To address small object detection in complex environments, Neri et al. (2025) proposed the integration of a CASAFF module to adaptively fuse channel and spatial features, achieving an improvement of up to 1.7% in mAP. With the same objective of enhancing multi-scale feature representation, Wang et al. (2025) incorporated a spatial pyramid pooling layer for multi-scale feature aggregation. Lightweight detectors for small objects, such as those proposed by Yu et al. (2024) and Jobaer et al. (2025), have been introduced to tackle field fruit recognition. Even so, accurate identification remains difficult owing to the extremely small pixel footprints and low resolution of the targets. In response to this limitation, Zhu Y. et al. (2025) added a fourth layer for small target detection and incorporated deformable convolution, achieving a grape picking rate of 87.4%. Similarly, Xiang et al. (2025) incorporated an additional small-target detection layer and integrated a simple parameter-free attention module to achieve precise weed localization. Incorporating dedicated small-target detection layers has been shown to improve accuracy (Chen et al., 2025). A drawback, however, is that these layers frequently inflate model complexity and computational cost. With the rapid advancement of Transformer models in computer vision, Miri et al. (2025) systematically investigated and categorized their performance advantages in SOD tasks. Building upon this foundation, Tian et al. (2025) combined a Mobile Vision Transformer architecture with a small-target detection layer to both maintain detection accuracy for small alfalfa seed pods and reduce model complexity. Crucially, these small-object detection methods were not designed to handle the near-color background confusion that is common in fruit orchard scenes.

Fruit recognition in near-color backgrounds. Accurate fruit identification in complex agricultural environments was crucial for vision-based harvesting robots (Zhang et al., 2025; Keshavarzi et al., 2025). For flat jujube, the color difference between ripe (yellowish-green) and unripe (chlorophyll-green) fruits during the harvesting period is minimal (Li et al., 2022). This results not only in low chromatic contrast against the background but also causes fruits at different maturity stages to be prone to mixing. Accordingly, Yang et al. (2024) developed an improved model based on YOLOv5 and GhostNet architectures using green fresh walnuts as the experimental subject, which achieved a 1.1% increase in mAP. Due to the reduced efficiency of selective harvesting (Zhu K. et al., 2025) by harvesting robot in low-color-contrast backgrounds, Sapkota et al. (2024) developed an instance segmentation method based on YOLOv8 and Mask R-CNN for immature green apples, achieving a accuracy of 93%. Similarly, Jia et al. (2025) proposed a foveabox-based boundary-aware segmentation model along with a boundary recovery module to mitigate boundary loss in low-color-contrast backgrounds, achieving a mean segmentation accuracy of 60.7%. Furthermore, some researchers employed attention mechanisms (Rahman et al., 2025) to enhance feature extraction capability in low-color-contrast backgrounds. For instance, Du et al. (2024) incorporated the convolutional block attention module to improve the acquisition of both channel and spatial attention information, achieving a single-class accuracy of 98.96%. However, conventional attention mechanisms offered limited improvement in feature extraction under low-color-contrast conditions, and insufficient attention was paid to edge information of irregularly shaped (Zhang et al., 2025; Keshavarzi et al., 2025; Saleh et al., 2025; Wiafe et al., 2025) fruits. Therefore, Liu et al. (2025) proposed a triple-branch attention mechanism combined with self-calibrating group convolution to enhance the extraction of tea shoot features and the capture of contextual information. Chen et al. (2024) introduced a selective kernel attention mechanism to improve the extraction of morphological features from irregularly shaped objects, achieving an mAP of 94.43%. Nevertheless, these near-color recognition methods largely neglect the challenge of detecting small objects and are typically not optimized for resource-constrained edge deployment.

Edge agricultural devices, such as harvesting robots and agricultural drones, are typically constrained by limited computational resources, and power consumption, which prevents the direct deployment of large-scale, highly complex models. To address these challenges, a growing number of researchers are focusing on the development of lightweight algorithms (Xu et al., 2025; Hu et al., 2025), aiming to achieve efficient visual perception with fewer parameters and lower computational costs. Therefore, there exists a pressing need for lightweight detection algorithms capable of recognizing small-sized fruits against near-color backgrounds to enhance the performance of automated fruit harvesting.

To address these task-specific challenges, we propose CFRDet. Specifically:

1. Mish activation preserves weak gradients, preventing small-target features from vanishing in lightweight networks.
2. The parameter-free ASAM suppresses near-color background interference without increasing parameters.
3. A small target detection layer handles the small clustered targets (small target challenge) by recovering spatial details.
4. MIRB heads ensure that the added computational cost (lightweight challenge) remains acceptable for field deployment.

Together, they form a collaborative chain of “feature preservation, background suppression, detail recovery, low-cost computation.”

## 2 Materials and methods

### 2.1 Preparation of the dataset

**Figure 1:**
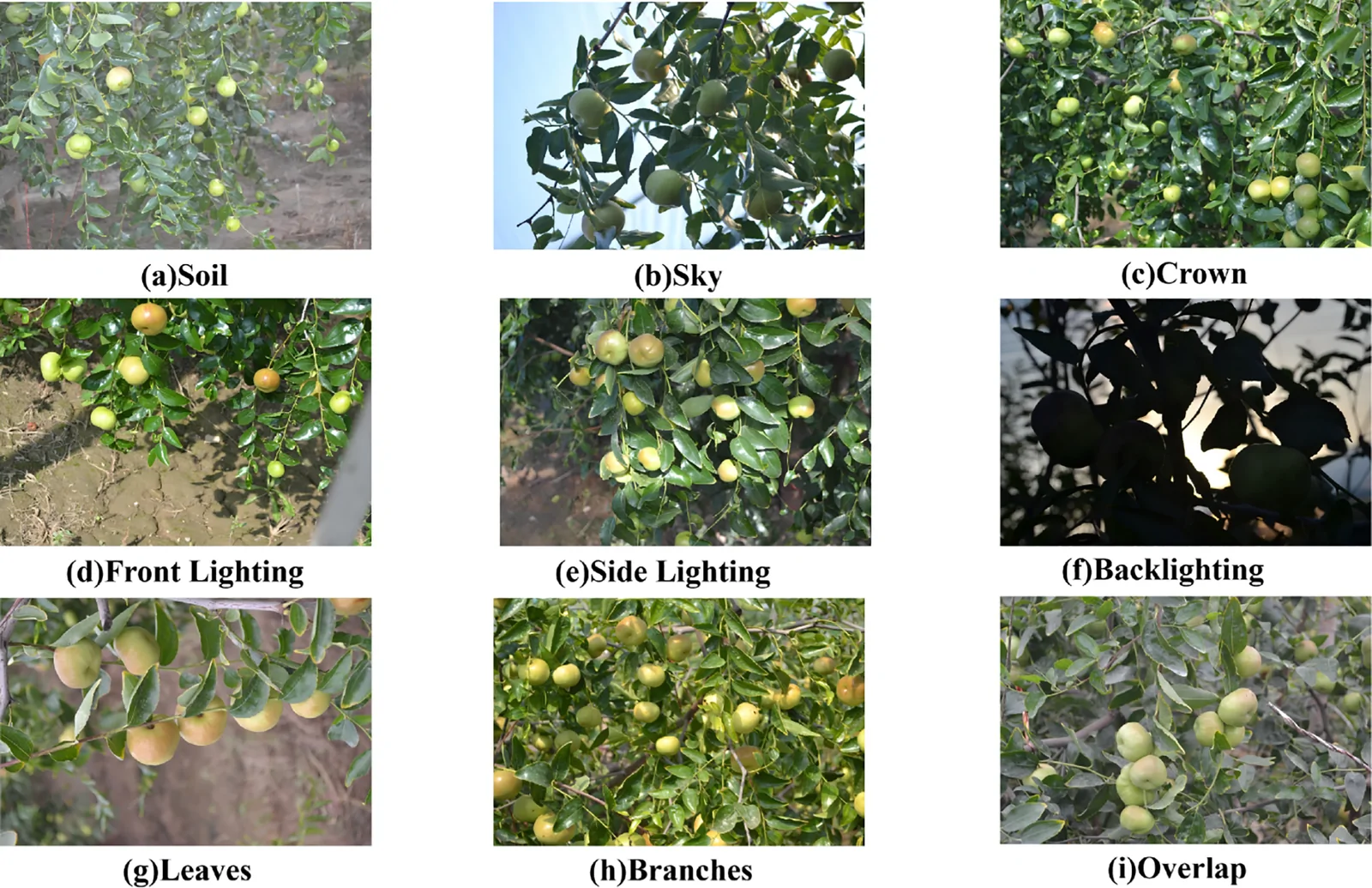
Some representative samples from the data set. **(a)** Soil. **(b)** Sky. **(c)** Crown. **(d)** Front Lighting. **(e)** Side Lighting. **(f)** Backlighting. **(g)** Leaves. **(h)** Branches. **(i)** Overlap.

In order to concurrently meet the requirements for handling small object detection task and distinguishing between similar colors, this study specifically constructed a self-built flat jujube datasets. The experimental orchards were located in Linyi County, Yuncheng City, and Taigu District, Jinzhong City, both in Shanxi Province, China. Image acquisition was carried out from July 15 to September 5, 2024, using a Nikon (Nikon Corporation, Tokyo, Japan) D3200 DSLR camera and an Apple smartphone. The images were captured at a distance of 50–80 cm from the target fruit. To enhance the model’s adaptability to varying image specifications, two resolutions—3456×2304 and 1920×1080 pixels—were included during data collection. To better replicate complex agricultural conditions, data were collected under varying scenarios including different types of occlusions (e.g., leaves, branches, and fruit overlap), background (soil, sky, crown), and lighting conditions (front, back, and side lighting). Representative samples of the acquired images are presented in **Figure 1**.

Based on the agronomic standards and harvesting requirements for flat jujubes at maturity, as well as practical insights from fruit growers, fruits during the harvesting period are categorized into two types: ripe fruits and unripe fruits. The mature and immature fruits exhibit yellowish-green and leaf-green coloration, respectively, both of which blend closely with the background foliage, thereby increasing the difficulty of visual identification. All annotations were manually performed using the LabelImg tool. The dataset was then randomly partitioned into training, validation, and test sets in an 8:1:1 ratio. The specific data are shown in **Table 1**.

**Table 1:** Distribution of data sets.

Data set	Image	Label		
		Ripe	Unripe	Total
Training set	8536	37095	20897	57992
Verification set	1067	4684	2786	7470
Test set	1067	6029	2843	8872
Total	10670	47808	26526	74334

In addition, to evaluate the generalization capability of the proposed method, a persimmon dataset was constructed, as persimmons also present a pronounced near-color detection difficulty in orchard environments. The images were collected in Wanrong County, Yuncheng City, Shanxi Province, over two periods: October 7–15, 2022 and October 13–18, 2024. Both a Honor smartphone and a Nikon D3100 DSLR camera were used for multi-angle photography, covering a wide range of agricultural scenarios, including varying illumination conditions, diverse background elements, and different degrees of occlusion. In total, 8518 images were acquired, with original resolutions ranging from 512×341 to 3456×2304 pixels, and all images were uniformly resized to 640×640 for model input. The dataset was randomly divided into training, validation, and test sets at a ratio of 8:1:1, respectively. The fruits in the images were manually annotated with bounding boxes using LabelImg by two annotators under the supervision of an agricultural expert, and all annotations were cross-checked to ensure consistency.

### 2.2 The overall architecture of CFRDet

#### 2.2.1.Activation function

Activation functions refer to pointwise nonlinear operations in neural networks that incorporate nonlinearity into the transformation of inputs. These functions determine the activation patterns of neurons, thereby influencing both the model’s representational capacity and gradient flow during training. In contrast to early activation functions such as Sigmoid and TanH, the Rectified Linear Unit (ReLU) has gained prominence due to its superior generalization capability and significantly accelerated convergence rate.

However, ReLU still suffers from several limitations, among which the most widely recognized is the “Dying ReLU” phenomenon: when inputs are negative, they are mapped to zero, resulting in vanishing gradients during training. Accordingly, numerous improved variants (ReLU6, AReLU, PReLU, RReLU, and Leaky ReLU) of ReLU have been proposed by researchers, each designed to address specific deficiencies. Furthermore, the Swish function, characterized by its smooth and continuous nature, demonstrates superior information propagation and enhanced robustness in deep neural architectures. Building on this, the Mish function represents a self-regularized non-monotonic activation function, its mathematical definition is given by Equation 1.

(1) $$f(x)=x\cdot \tanh(softplus(x))=x\cdot \tanh(\ln(1+e^{x}))$$

Where x is the input scalar (pre-activation), f(x) is the output scalar, e is Euler’s number, ln(·) denotes the natural logarithm.

**Figure 2:**
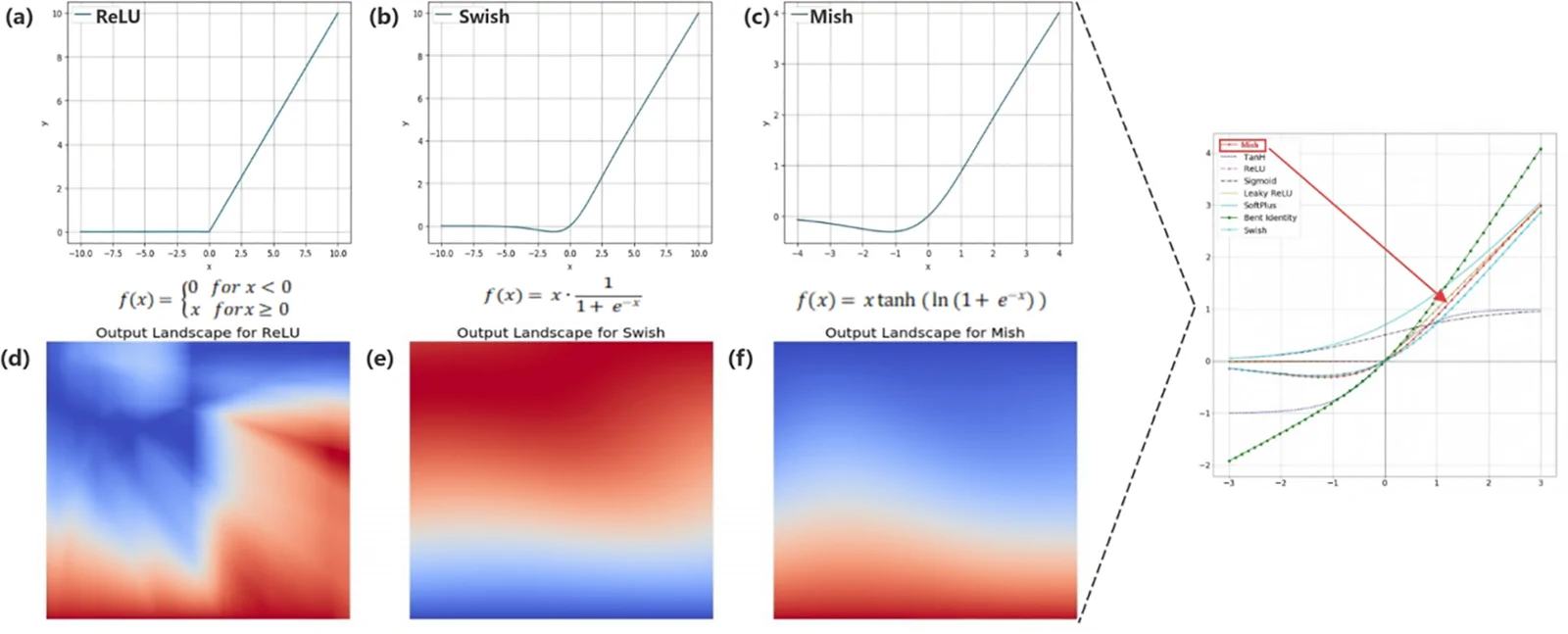
Comparison of various activation functions. **(a–c)**: Graphical representations of the ReLU, Swish, and Mish activation functions. **(d–f)**: the output landscapes of neural networks employing the three activation functions.

Mish incorporates the self-gating property of the Swish activation function, in which the ungated input is multiplied by the output of a nonlinear activation. By preserving a small amount of negative information, it eliminates the preconditions required for the Dying ReLU phenomenon. This characteristic contributes to enhanced expressive power and improved efficiency of information flow. Unlike ReLU, Mish is continuously differentiable, a property that prevents the occurrence of singular points and thereby reduces undesirable side effects during gradient-based optimization. A comparative of the outputs from the three activation functions is presented in **Figure 2**.

As illustrated in **Figure 2**, which compares the output landscapes of neural networks employing ReLU, Swish, and Mish activation functions, ReLU exhibits the most pronounced sharp transitions. These discontinuities are partially reduced in Swish, while Mish produces the smoothest (**Figures 2d–f**) output landscape with the fewest irregularities. Mish activation function is unbounded above, which helps mitigate vanishing gradients caused by saturation. At the same time, its bounded below property induces a strong regularization effect (**Figure 2c**).

In the study of in-field agricultural object detection, the Mish activation function enables the network to learn and represent highly complex nonlinear relationships inherent in field environments, thereby facilitating the construction of sophisticated decision boundaries. This capability enhances the robustness of small object recognition against various disturbances. More importantly, Mish promotes feature separation and sparsity, effectively suppressing irrelevant background noise. This property is particularly beneficial for improving recognition performance in scenarios involving similarly colored objects.

#### 2.2.2 Area-attention similarity-aware module

Existing attention modules exhibit limitations in capturing attention weights across both channel and spatial dimensions, and often entail complex structures that lead to increased computational costs. A representative example is the Squeeze-and-Excitation (SE) attention mechanism, which performs feature recalibration by weighting different channels. On this basis, various extended attention mechanisms have been developed, such as the Channel Prior Convolutional Attention (CPCA) and Spatial and Channel Synergistic Attention (SCSA).

**Figure 3:**
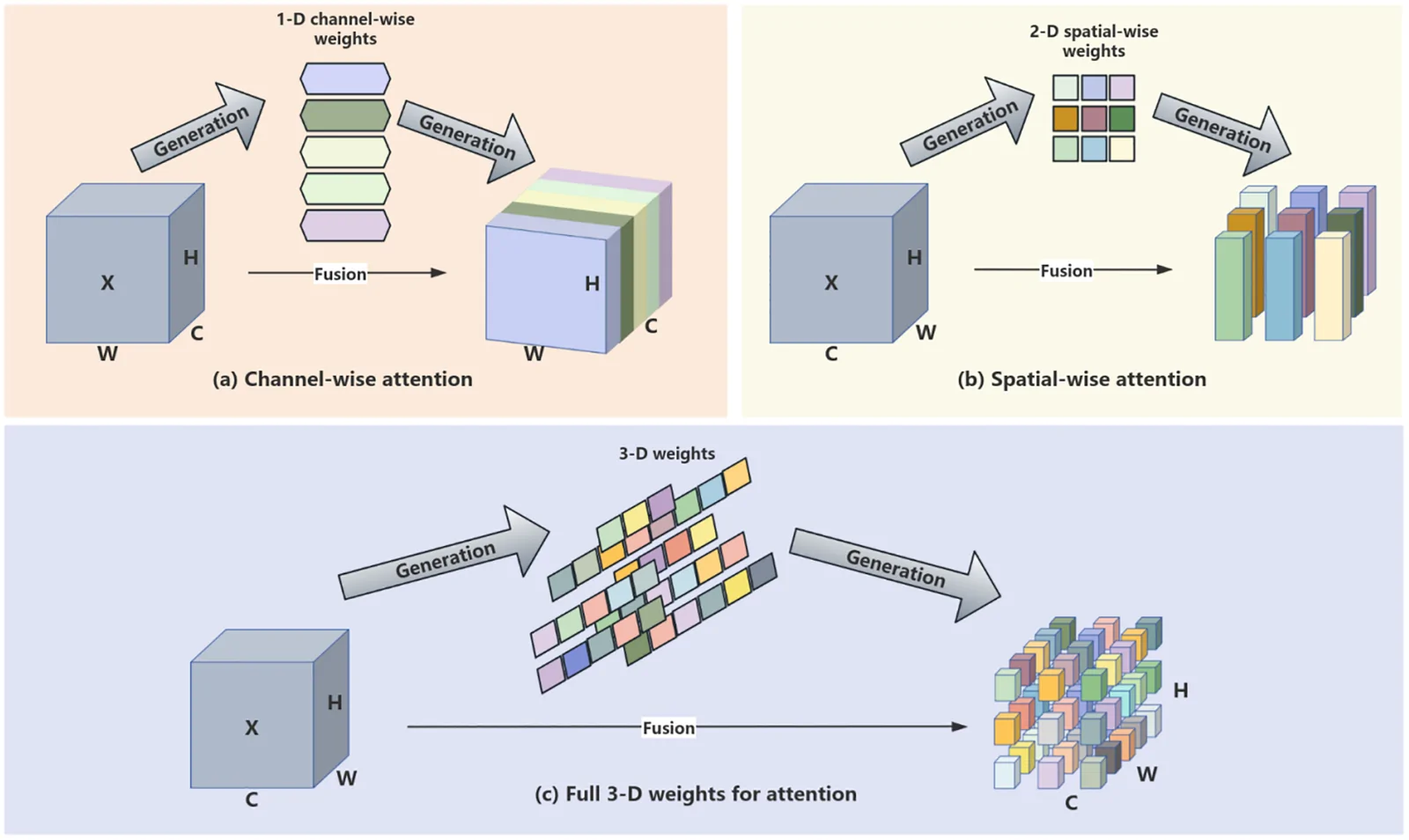
Comparison of different types of attention mechanisms. **(a)**: Channel-wise attention. **(b)**: Spatial-wise attention. **(c)**: Full 3-D weights for attention.

However, these methods share a common limitation: the inability to effectively compute fully three-dimensional attention weights. That is, they either treat all features within a channel equally or process all features at the same spatial location uniformly, rather than capturing joint channel-spatial dependencies. To address these issues, we introduce ASAM, a parameter-free attention mechanism that distinguishes itself from modules such as SE and CBAM, which rely on additional network layers to generate attention maps. The specific differences are shown in **Figure 3**.

As illustrated above, most existing attention mechanisms generate 1-D (**Figure 3a**) or 2-D (**Figure 3b**) weights from the input features X which are then expanded to form either channel-wise or spatial attention maps. This approach limits their ability to learn highly discriminative representations. In contrast, the ASAM mechanism directly computes a 3-D weight matrix (**Figure 3c**), thereby enhancing feature extraction—particularly in distinguishing objects from backgrounds with similar colors.

The core idea of ASAM is to estimate the importance of each neuron by defining an energy function: neurons with lower energy are considered more important. Based on this principle, a concise and analytical formulation for attention weights is directly derived to enhance feature representation. Specifically, to make the network learn more discriminative neurons, Yang et al. (2021) proposed to directly infer 3-D weights (i.e., considering both spatial and channel dimensions) from current neurons and then in turn refine those neurons. Notably, most of the operators employed in this module are derived directly from the optimization process of the energy function, thereby eliminating the need for additional complex components. As a result, it introduces no extra parameters or computational overhead. ASAM is grounded in well-established neuroscience principles, particularly the phenomenon of spatial suppression observed in mammalian brains, where active neurons suppress the activity of their neighbors. This mechanism formulates the weight generation process as an energy function, as specified in Equations 2 and 3.

(2) $$e_{t}\left(w_{t},b_{t},y,x_{i}\right)=\left(y_{t}-{\hat{t}}^{2}\right)+\frac{1}{M-1}\sum_{i=1}^{M-1}\left(y_{0}-{\hat{x}}_{i}^{2}\right)$$

(3) $$\hat{t}=w_{t}t+b_{t},\;{\hat{x}}_{i}=w_{i}x_{i}+b_{t}$$

Here, Equation 3 are linear transforms of t and x_i_, where t and x_i_ are the target neuron and other neurons in a single channel of the input feature X∈R^C×H×W^. i is index over spatial dimension and M = H×W is the number of neurons on that channel. w_t_ and b_t_ are weight and bias the transform.

By minimizing this function, Equation 2 is equivalent to seeking linear separability between the target neuron t and all other neurons within the same channel. To simplify the formulation, binary labels (i.e., 1 and -1) are introduced for y_t_ and y_o_, and a regularization term is incorporated into Equation 2. Substituting Equation 3 into Equation 2 yields the minimal energy function, given in Equation 4.

(4) $$e_{t}^{*}=\frac{4\left(\partial^{2}+\lambda \right)}{{\left(t-\hat{\mu }\right)}^{2}+2\sigma^{2}+2\lambda }$$

(5) $$\hat{\mu }=\frac{\sum_{i=1}^{M}x_{i}}{M},\;\partial^{2}=\frac{\sum_{i=1}^{M}{\left(x_{i}+\hat{\mu }\right)}^{2}}{M}$$

Here, the mean and variance computed over all neurons except neuron t are given by Equation 5.

The equation above indicates that a lower energy value $e_{t}^{*}$ for neuron t corresponds to higher distinctiveness from surrounding neurons, implying a more critical role in visual processing. ASAM operates on individual neurons, incorporates this linear separability into an end-to-end learning framework, and employs a scaling operation during feature refinement rather than simple addition.

#### 2.2.3 Small target detection layer

In complex agricultural environments, medium-sized fruits are often obscured by leaves, branches, and other obstructions, causing them to appear as smaller, fragmented objects in images. Furthermore, due to long shooting distances or wide-angle lens settings, fruits may occupy very few pixels in the image. These factors make it particularly challenging to detect such small targets against cluttered backgrounds. Most current object detection algorithms, whether single-stage or two-stage, employ multiple downsampling operations during feature extraction. Each downsampling step leads to the loss of fine-grained spatial information. For instance, a small target measuring 20×20 pixels may occupy less than one pixel on the feature map after 32 downsampling. As a result, its feature information becomes almost entirely submerged, making effective detection infeasible.

**Figure 4:**
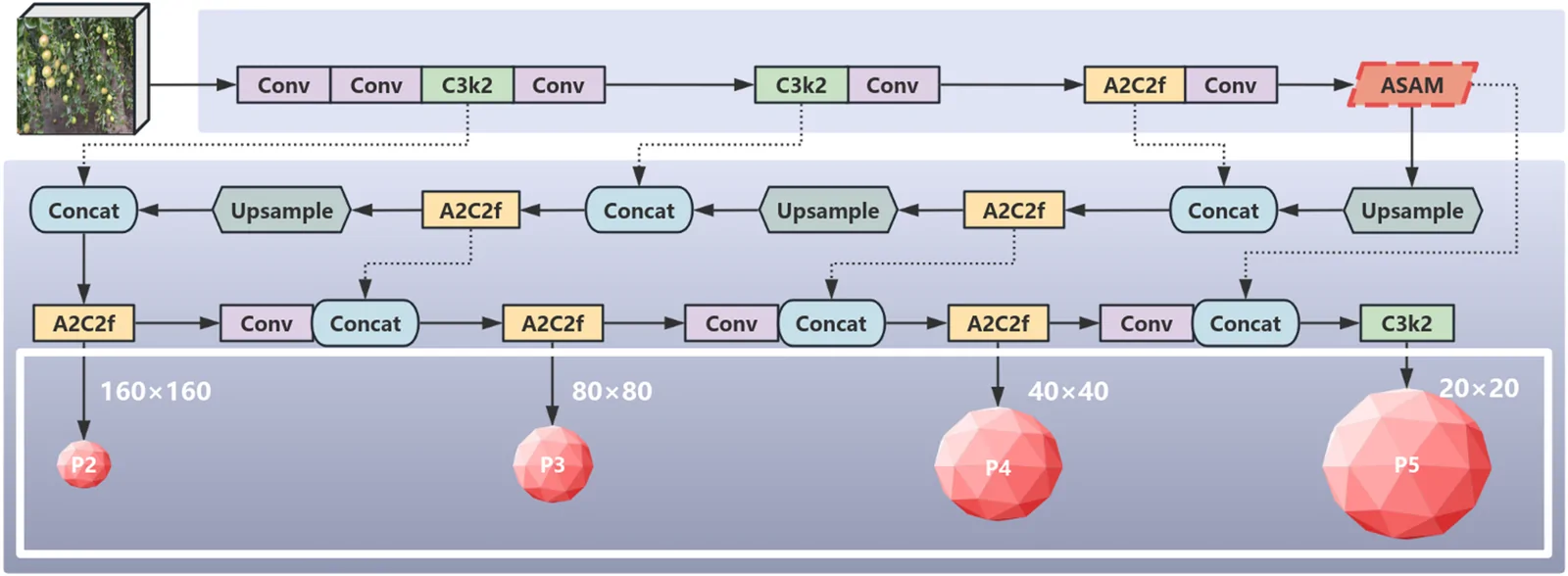
The structural diagram of the improved network.

The addition of a small-target detection layer is essentially a strategy for multi-scale feature fusion, which can be applied prior to any downsampling stage in the backbone network. During feature extraction, the backbone network produces both shallow and deep feature maps: the former exhibit weak semantics but high resolution and rich detail, while the latter contain strong semantic information but suffer from low resolution and significant detail loss. Our approach specifically aims to extract higher-resolution shallow feature maps (2th layer) from the backbone network and integrate them with deep feature maps (17th layer) via upsampling in the neck network, thereby yielding a new detection layer (18th layer) that combines strong semantic information with high-resolution detail, specifically designed for detecting small-sized objects. The specific structure is shown in **Figure 4**.

In object detection, effectively capturing the features of smaller targets necessitates the use of feature maps with higher resolution. Within the white solid box in **Figure 4**, the P5/32 layer—obtained through the most downsampling operations—exhibits the lowest spatial resolution, yet the strongest semantic information and the largest receptive field, making it suitable for detecting large objects. In contrast, the P2/4 layer is a newly incorporated detection layer dedicated to small targets, which boasts the highest spatial resolution along with abundant detailed information, and is particularly suited for detecting smaller objects. Through dedicated convolutional and upsampling operations, this feature layer captures finer-grained characteristics of small objects and effectively integrates with features from other layers, thereby enhancing both the detection precision and recall rate for small targets. This approach effectively addresses the limitations of the original model in detecting small objects, leading to significantly improved performance in scenarios containing numerous small targets.

#### 2.2.4 Mobile inverted residual bottleneck

**Figure 5:**
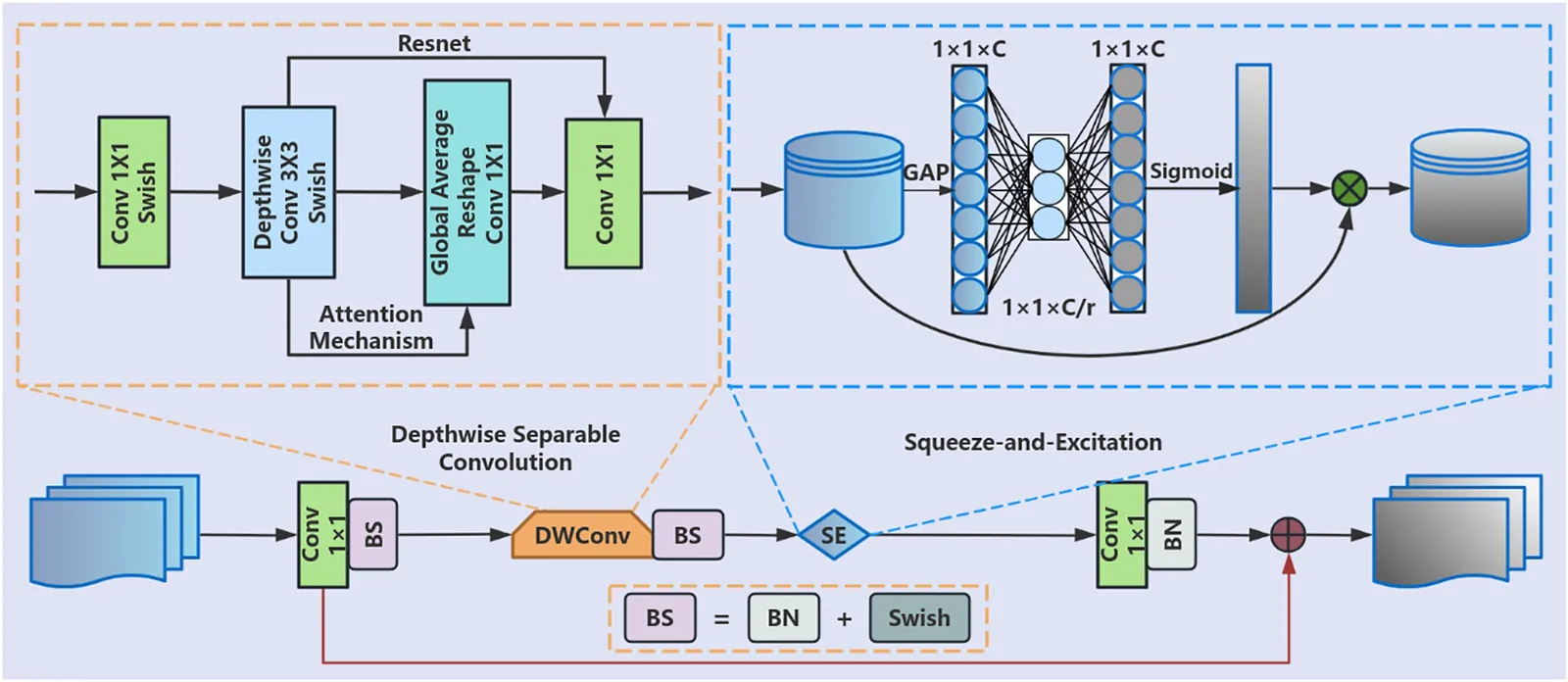
Structure diagram of MIRB.

Scaling up ConvNets is widely used to achieve better accuracy. Conventional approaches typically expand the network along only one dimension, such as depth (layers), width (channels), or input image resolution. In contrast, EfficientNet-B0 (Salka et al., 2025) represents a compound scaling approach that simultaneously expands the network along depth, width, and resolution dimensions using a fixed scaling ratio. This uniform scaling strategy ensures a balanced allocation of computational resources across these dimensions, with its core module being the Mobile Inverted Residual Bottleneck (MIRB) incorporating the SE optimization, as detailed in **Figure 5**.

The rationale behind this method lies in the fact that larger input images require a deeper network to increase the receptive field size, while also needing more channels to capture fine-grained patterns present in higher-resolution images. The MIRB module operates through a sequence of well-defined steps. Firstly, a pointwise convolution (1×1 convolution) is used to increase the channel count, expanding the feature dimensionality. This allows the subsequent depthwise convolution (DWConv) to operate in a higher-dimensional space, thereby enhancing its capacity to capture richer representations. Secondly, efficient feature extraction is achieved and the computational cost of conventional convolution is reduced by using DWConv (orange dashed line in **Figure 5**) with a kernel size of 3×3. This operation is the primary source of computational savings, as it eliminates the need for the joint and costly processing of both spatial and channel dimensions. Subsequently, a SE attention mechanism adaptively re-calibrates channel-wise feature responses to enhance the representation capability of important features. Finally, the expanded high-dimensional features are projected back to a low-dimensional space, forming a bottleneck structure, and a skip connection (red solid line in **Figure 5**) is incorporated to avoid the issues of gradient vanishing and overfitting.

The resulting inverted residual bottleneck structure thus exhibits a spindle-shaped form (“small-large-small”), which stands in direct contrast to the hourglass configuration (“large-small-large”) of traditional residual blocks. This expansion-then-reduction design prevents information loss that would otherwise occur if residual connections were applied directly in a low-dimensional space. As a result, it more effectively preserves critical information throughout the network. The MIRB module offers high computational efficiency. The use of DWConv significantly reduces computational complexity compared to conventional convolution operations, while maintaining competitive feature extraction capabilities and lowering computational costs. These characteristics make it well-suited for deployment on resource-constrained devices. Furthermore, the incorporation of skip connections helps mitigate the risk of gradient vanishing and overfitting. In addition, MIRB exhibits strong representational capacity. By employing an inverted bottleneck structure that expands and subsequently reduces feature dimensions, along with the channel attention mechanism in the SE module, it enhances feature extraction and utilization, thereby improving model robustness.

### 2.3.Principle of collaborative design

The three challenges (color-similar background, small targets, lightweight) are not independent. A small target in a color-similar background produces extremely weak feature responses, which are easily lost in lightweight networks that sacrifice capacity for speed. To address the limitations of detecting small targets and distinguishing objects against similarly colored backgrounds in field environments for flat jujubes, and to accommodate the future deployment of lightweight models, this paper proposes an improved lightweight algorithm based on YOLOv12n (Tian et al., 2026).

**Figure 6:**
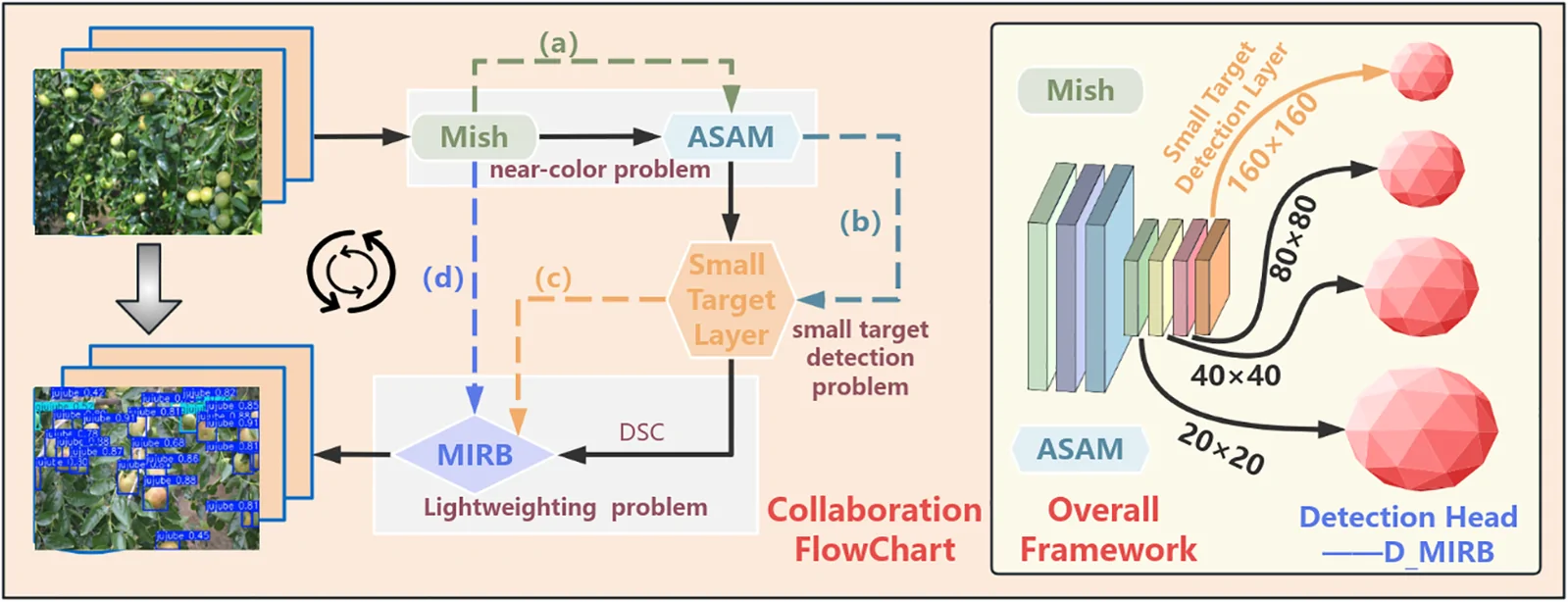
Collaborative flow of the four proposed components. The data flows from input to output via the backbone (with Mish), ASAM, small-target detection layer, and MIRB-based head. Dashed arrows indicate principle-level synergies: **(a)** Mish prevents vanishing gradients for weak tiny-object features, providing a valid input space for ASAM; **(b)** ASAM suppresses color-similar background noise without parameters, ensuring that the subsequent high-resolution feature map is not dominated by clutter; **(c)** The tiny-object detection layer recovers spatial details, but its high computational cost is offset by the MIRB head; **(d)** Mish also improves gradient backpropagation inside the depthwise convolutions of MIRB, mitigating the degradation typical of lightweight inverted residuals.

Firstly, to promote feature separability and sparsity, thereby boosting the model’s performance in identifying fruits within near-color backgrounds, the Mish activation function (the light green region in **Figure 6**) was adopted to refine the basline network. Secondly, to enhance the detection performance of the model in near-color backgrounds while adhering to the principle of minimizing additional parameters and computational cost, a parameter-free ASAM attention module (the light blue region in **Figure 6**) was introduced to improve the discriminative capability against targets in such challenging scenarios. Furthermore, an additional detection layer (the orange text annotations in **Figure 6**) designed for small objects was incorporated into the baseline network to enhance the detection capability for such targets. Finally, to strengthen the multi-scale adaptability of the model after adding the small-object detection layer and to improve the detection head’s capacity for objects of various sizes, an MIRB-based detection head (the red polyhedral spherical in **Figure 6**) was integrated. The overall architecture of the proposed method is illustrated in **Figure 6**.

Thus, the four modules form a closed-loop solution to the triple conflicts (near-color background, tiny objects, and lightweight requirement). **Table 2** presents the comparative results of synergistic points.

**Table 2:** Point alignment comparison.

Synergistic points	Relationship	Mechanism	Resolved conflict
Mish → ASAM	Weak Feature Purification Chain	Mish retains minor differences → ASAM calculates more accurate attention	Weak features against near-color background & requiring discriminability
ASAM → Small target layer	Noise-reduction high-resolution features	High-resolution features after noise reduction → Avoid amplification of background noise	High-resolution images are prone to noise & small targets require clean details
Small target layer → MIRB	Low-cost high-resolution detection	MIRB achieves high-resolution image processing with linear complexity → making small target layer computationally feasible	Small goals require big visuals & lightweight constraints
Mish → MIRB	Prevent disappearance of MIRB gradient	Mish improves deep convolutional gradient flow → prevents feature degradation within MIRB	Depth separable convolutional networks prone to gradient vanishing & networks requiring stable training

→ denotes the synergistic optimization between pairwise innovations.

## 3 Experimental results and discussion

The hardware specifications and experimental environment used in this study are shown in **Table 3**.

**Table 3:** Experimental environment configuration.

Hardware	Configure	Environment	Version
System	Windows 11 (64-bit)	Python	3.11.5
CPU	Intel Core U9-285K	PyTorch	2.8.0
GPU	NVIDIA RTX 5090 (32G)	PyCharm	2025.2.6
RAM	64 GB	CUDA	CUDA 12.8
Hard-disk	3 TB	CUDNN	9.8.0

Based on the available hardware resources and model parameters, the batch size was set to 128, and the initial learning rate was set to 0.01. The momentum factor, weight decay, and number of training epochs were configured as 0.9, 0.0005, and 100, respectively. The dataset used for training was a custom-built flat jujube image dataset, with input images resized to 640×640 pixels.

### 3.1 Experiment on activation function optimization

A suitable activation function can mitigate vanishing gradients and enable more stable feature learning, providing a stronger foundation for subsequent architectural improvements. Using the default SiLU (Swish) as the baseline, we compared eight activation functions: ReLU and five of its variants, Hard Swish, and Mish. Since none of these alter the model’s parameter count or GFLOPs, the comparison focused solely on training performance, with the results summarized in **Table 4**.

**Table 4:** Performance comparison of different activation functions.

Activation function	P (%)	R (%)	mAP(%)	mAP@0.5:0.95(%)	F1 (%)	Detection time (ms)			
						Pre- treatment	Inference time	Post- treatment	Total time
SiLU(Swish)	90.2	86.4	95.0	77.8	88	0.1	0.3	1.1	1.5
Hard Swish	90.8	85.9	94.8	77.3	88	0.1	0.3	1.2	1.6
Mish	90.5	88.3	95.4	79.2	89	0.1	0.4	1.0	1.5
ReLU	89.0	86.3	94.0	76.1	88	0.1	0.3	1.0	1.4
ReLU6	89.0	86.2	94.3	76.7	88	0.1	0.3	1.1	1.5
AReLU	91.1	87.2	95.2	78.3	89	0.1	0.6	0.9	1.6
PReLU	90.4	86.5	94.9	77.4	88	0.1	0.3	1.1	1.5
RReLU	90.1	86.8	94.9	77.8	88	0.1	0.4	1.0	1.5
Leaky ReLU	90.0	85.7	94.1	76.4	88	0.1	0.3	1.1	1.5

**Figure 7:**
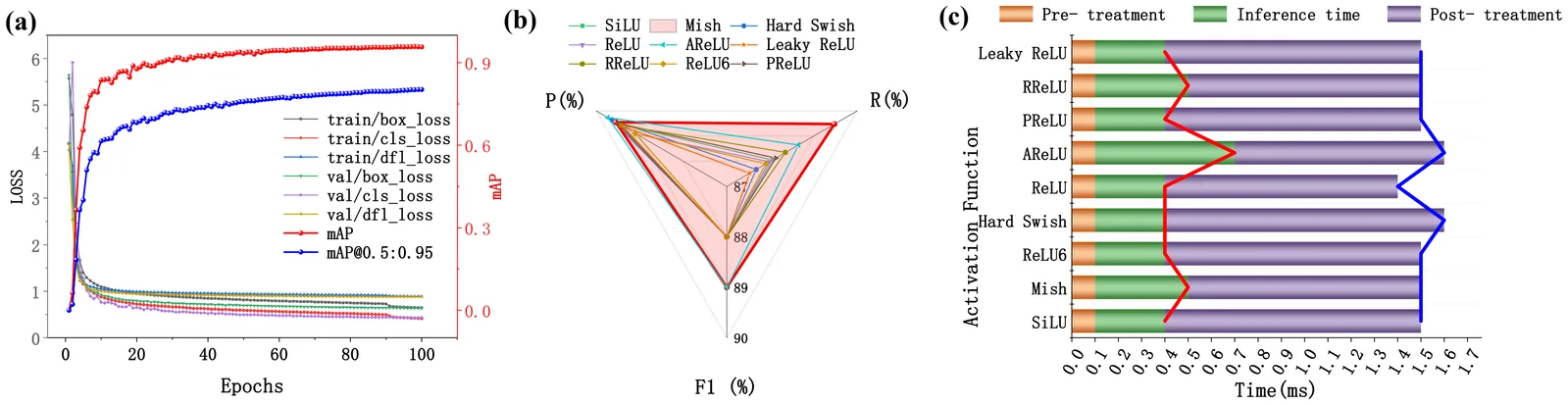
The effect of different activation functions. **(a)**The training performance of the Mish activation function. **(b)** Comparative accuracy of nine activation functions. **(c)** Comparative inference time of the nine activation functions.

Among the ReLU-based variants, only AReLU improved accuracy, but this came at the cost of higher inference time, a trade-off consistent with Medani et al. (2025) and attributable to its per-channel learnable parameters. Hard Swish, a piecewise-linear approximation of Swish, decreased mAP and mAP@0.5:0.95 by 0.2 and 0.5 percentage points, respectively, and added 0.1 ms of post-processing overhead. The most plausible explanation for this behavior lies in its piecewise-linear formulation, which introduces conditional branches that may incur branch prediction penalties during post-processing. A detailed comparison is provided in **Figure 7**. Although Swish showed clear advantages in this agricultural setting, Mish delivered further gains.

Swish itself, with its smooth and non-monotonic profile, facilitated finer feature learning and offered clear advantages for discriminating subtle color differences in agricultural scenes. However, Mish delivered further gains. By introducing stronger nonlinearity, Mish improved gradient flow and promoted feature sparsity, effectively suppressing background interference under near-color conditions. It promotes feature separation and sparsity, suppresses irrelevant background information, and improves detection accuracy (**Figures 7a, b**) under challenging color conditions. Experimental results demonstrated that replacing the original activation function with Mish yielded increases in mAP, mAP@0.5:0.95, and F1-score by 0.4, 1.4, and 1.0 percentage points, respectively, without altering the total detection time(**Figure 7c**). These improvements proved particularly valuable for capturing the weak features of small fruit targets. Consequently, we adopt Mish as the activation function throughout the proposed CFRDet.

### 3.2 Experimental performance of ASAM attention mechanism without parameters

Building upon a well-established foundational feature extractor (named M-YOLO, as detailed in the preceding section), an attention mechanism was introduced to further improve feature quality. The attention mechanism guided the network to emphasize more critical regions, thereby enhancing the discriminative capacity of the features. To strengthen feature extraction performance with the dual objective of facilitating subsequent lightweight deployment, this study employed 4 lightweight attention modules for extensive comparative experiments, including ASAM, Channel Prior Convolutional Attention (CPCA, Huang et al. (2024)), Multi-Scale Convolutional Attention (MSCA), and Large Separable Kernel Attention (LSKA). The highest accuracy achieved by each module and its combination scheme across different architectural locations was selected as the experimental outcome, as showed in **Table 5**:

**Table 5:** Performance comparison of different attention mechanisms.

Attention mechanism	P (%)	R (%)	mAP(%)	mAP@0.5:0.95 (%)	F1 (%)	Parameters	FLOPs(G)	Model size(MB)	Layer
M-YOLO	90.5	88.3	95.4	79.2	89	2520054	6	5.4	497
+CPCA	90.5	87.8	95.2	79.3	89	2677046	6.7	5.7	510
+LSKA	87.8	86.0	93.6	75.7	87	1758342	4.5	3.7	277
+MSCA	90.9	88.2	95.6	79.5	89	2643958	6.5	5.7	506
+ASAM	91.4	88.8	95.9	80.4	90	2550006	6.5	5.5	499
+ASAM+CPCA	90.3	84.4	95.5	80.3	89	2677046	6.7	5.7	512
+ASAM+LSKA	89.7	87.4	94.9	78.9	89	1758342	4.5	3.7	279
+ASAM+MSCA	91.1	89.0	95.8	80.2	90	2643958	6.5	5.6	508
+MSCA+LSKA	89.4	87.4	94.5	77.8	88	1852294	4.6	3.9	286
+MSCA+CPCA	90.5	87.8	95.2	79.3	89	2770998	6.8	5.9	519
+CPCA+LSKA	89.2	86.4	94.3	77.5	88	1885382	4.7	4.0	290

The symbol “+” denotes the replacement of a single module on the current baseline network.

**Figure 8:**
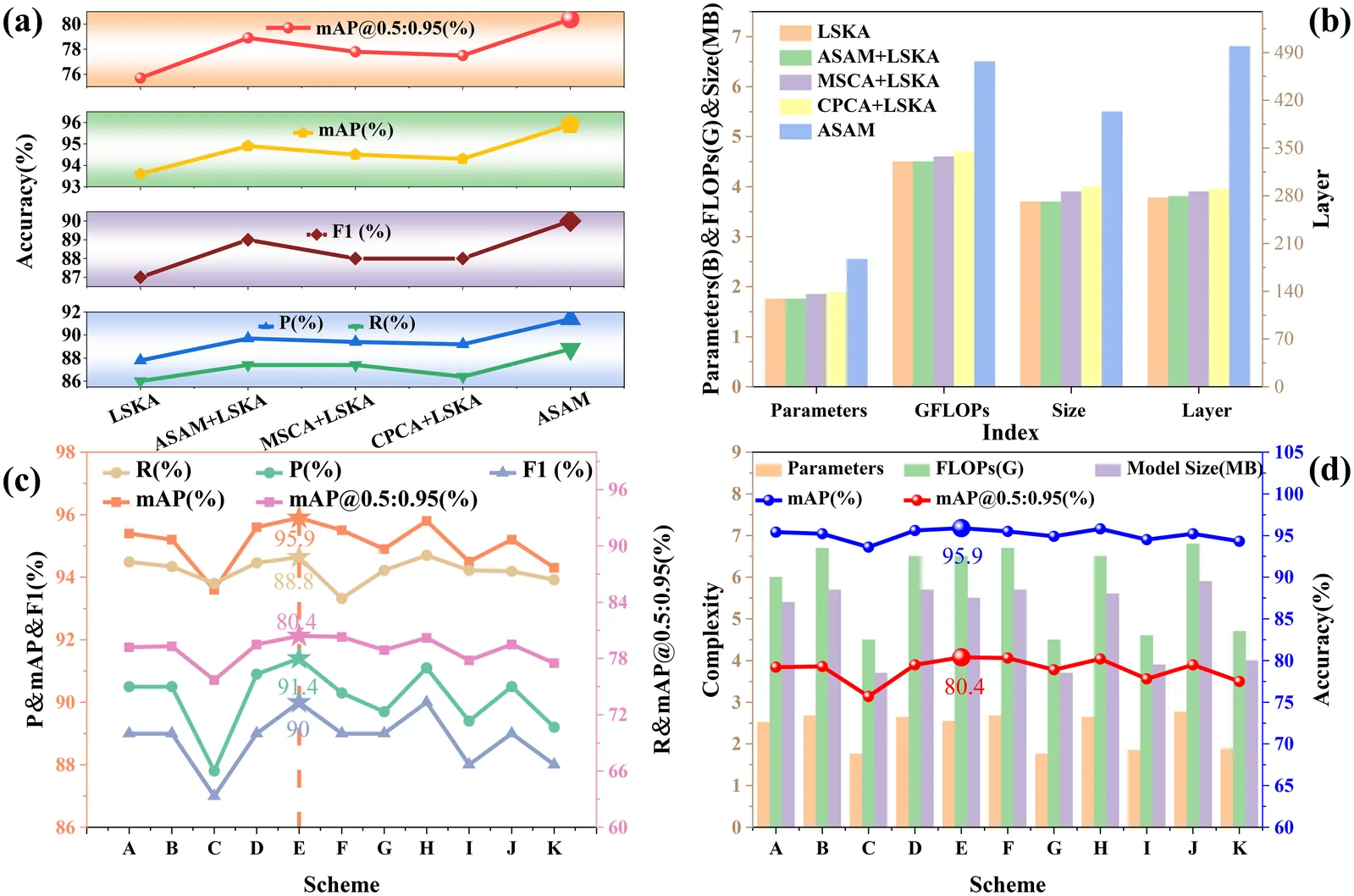
The improvement effect of lightweight attention mechanism. **(a)** Impact of LSKA versus ASAM on model accuracy. **(b)** Impact of LSKA versus ASAM on model complexity. **(c)** Detection Performance: Baseline Network *vs*. 10 Attention Mechanisms. **(d)** Comparison of Accuracy Metrics across Different Attention Mechanisms.

As shown in the **Table 5**, the single ASAM and the combined ASAM+MSCA schemes achieved mAP values of 95.9% and 95.8%, and mAP@0.5:0.95 values of 80.4% and 80.2%, respectively, both ranking among the top two. However, the latter exhibited increases in parameter count (+3.8%), model size (+1.8%), and the number of network layers compared to the former, indicating that the single ASAM configuration was more efficient. The LSKA module was fundamentally a large-kernel depthwise convolution (Lau et al., 2024). Its lightweight advantage arose from replacing self-attention with depthwise separable convolution. However, precisely due to the enlarged receptive field and static convolution weights introduced by the large kernel, the module was unsuitable for tasks requiring fine-grained perception, such as small object detection, where it incurred substantial accuracy degradation (decreased of 1.8% and 3.5% in mAP and mAP@0.5:0.95). Detailed visual results are provided in **Figure 8**.

As shown in **Figures 8a, b**, although LSKA reduced model complexity, it incurred a significant degradation in accuracy; therefore, this scheme was excluded from further consideration. The CPCA module, which operates via decomposed coordinate pooling, exhibited heightened sensitivity to positional information. However, owing to its reliance on 1D global pooling, it lacked the capacity to process contextual information, resulting in a slight decline in detection accuracy. Moreover, the number of parameters, GFLOPs, and model size increased by 6.2%, 11.7%, and 5.6%, respectively. Similarly, the MSCA module, also based on channel attention, led to an increase in model complexity. Nevertheless, by incorporating dilated convolution, it enhanced multi-scale feature extraction and contextual fusion capabilities, which contributed to a modest improvement in accuracy (Guo et al., 2022). That said, its lightweight efficiency remained inferior to that of the parameter-free ASAM module.

Among the top ten best-performing improved schemes, scheme E (single ASAM, named MA-YOLO) achieved the highest scores across all accuracy metrics (**Figures 8c, d**). In addition, it ranked first in lightweight efficiency except for the LSKA module, demonstrating the best overall improvement performance. Its primary advantage lay in its derivation of a closed-form solution to an energy function, which introduced no additional parameters. By incorporating feature information from both spatial and channel dimensions, it effectively enhanced the recognition of low-contrast and small objects.

### 3.3 Multi-scale fusion improvement

Following the enhancement of foundational features through the incorporation of the Mish activation function and the ASAM attention mechanism in the preceding two sections, this study further improved the feature fusion architecture. As this structure plays a key role in multi-scale object detection and the target objects in this research are predominantly small, a dedicated detection layer for small objects was introduced in the neck network. For comparative purposes, an additional detection layer designed for large objects was also integrated and evaluated. In addition, the architectures of Hyper-YOLO, ASF-YOLO, and Gold-YOLO, which excel at capturing fine-grained features, were adopted for comparative evaluation. The corresponding experimental results are provided in **Table 6**.

**Table 6:** Adjustment of detection layer and improvement of neck network structure.

Scheme	AP(%)		mAP(%)	mAP@0.5:0.95(%)	F1 (%)	Parameters	FLOPs (G)	Size (MB)	Layer
	Ripe	Unripe							
MA-YOLO	97.2	94.6	95.9	80.4	90	2550006	6.5	5.5	499
+Small target layer	97.5	95.3	96.4	82.3	91	2714936	12.8	5.9	595
+Large target layer	97.2	94.7	95.9	81.0	90	3965688	6.4	8.4	710
+Hyper-YOLO	97.3	95.2	96.3	81.4	91	3171446	8.2	6.8	574
+ASF-YOLO	97.0	93.4	95.2	79.5	89	2881857	7.9	6.2	599
+Gold-YOLO	97.5	95.2	96.4	82.0	90	5617462	10.5	11.7	685

The symbol “+” denotes the replacement of a single module on the current baseline network.

**Figure 9:**
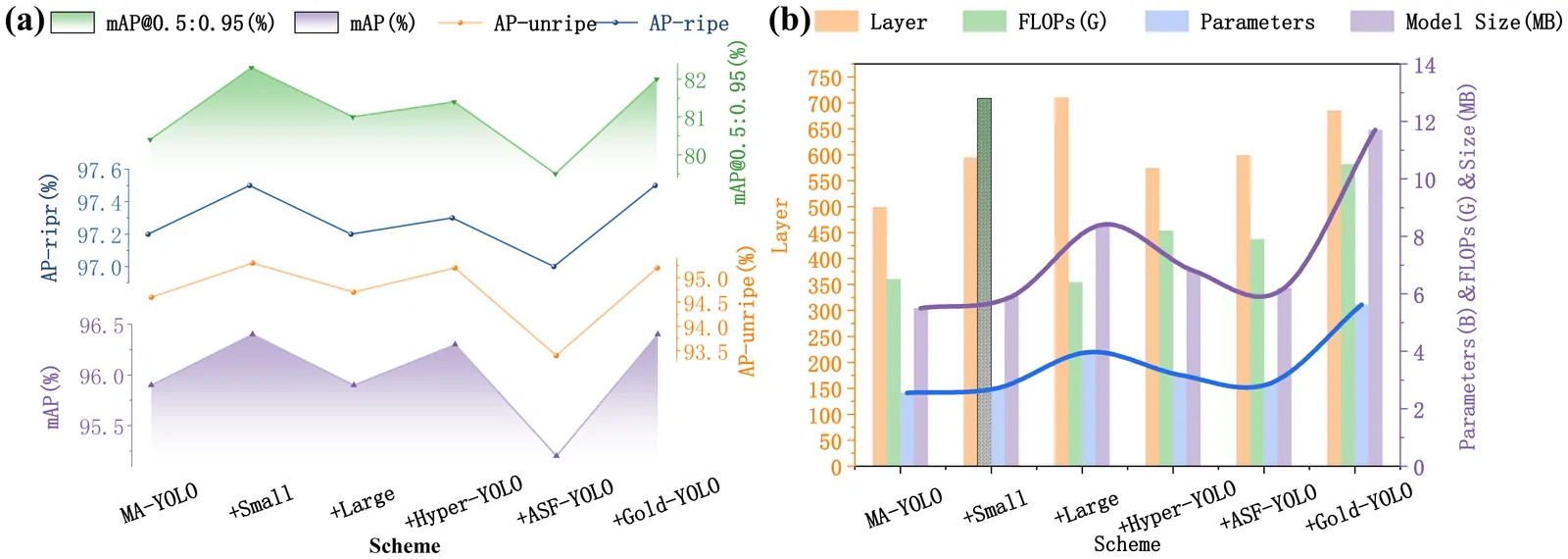
The influence of 5 improvement schemes on the performance of small target detection. **(a)** The influence of each scheme on model accuracy. **(b)** The influence of each scheme on model complexity.

As evidenced by the experimental results, among the five improved schemes, only the Small target layer and Gold-YOLO models achieved the highest mAP of 96.4%. Moreover, the former attained a superior mAP@0.5:0.95 of 82.3% (**Figure 9a**). Notably, the parameter count and model size of the S model were only 48.3% and 50.4% of those of the Gold-YOLO model, respectively, demonstrating its optimal overall performance. The Gold-YOLO network (Wang et al., 2023) benefited from the Gather-and-Distribute mechanism and the fusion module, which enhanced information interaction and flow across different feature levels. However, this architecture also significantly increased model complexity. Detailed visual comparisons are provided in **Figure 9**.

As shown in **Figure 9b**, an interesting yet important phenomenon was observed: the GFLOPs of the small-object detection layer increased sharply, while its model size remained almost unchanged. This discrepancy arises from the distinct computational principles underlying GFLOPs and model size. The former is highly sensitive to spatial dimensions (H×W) and operates on feature maps with high resolution but low channel numbers; whereas the latter depends strongly on the number of channels (Cin×Cout) and is associated with feature maps of low resolution yet high channel counts. The small-object detection layer processes high-resolution, low-channel-count feature maps, which explains why the GFLOPs exhibited a sharp increase while the number of parameters remained largely unchanged. Therefore, the small-target detection layer enhances the model by employing multi-scale feature fusion, which supplies feature maps integrating both high-resolution details and semantically rich information. This improvement (named MAS-YOLO) leads to a notable increase in detection accuracy for small objects, and greater robustness in complex agricultural settings.

### 3.4 Improvements in the detection head

To address the limitation of the sharp increase in GFLOPs after adding detection layers for small objects, we explored the integration of lightweight detection heads. Three alternative designs—MIRB, Conv2Former, and DynamicConv heads—were investigated to mitigate the computational overhead. The results are shown in **Table 7**.

**Table 7:** Improvements in the light weight detection head.

Scheme	AP(%)		P (%)	R (%)	mAP(%)	mAP@0.5:0.95 (%)	F1 (%)	Parameters	FLOPs(G)	Size (MB)
	Ripe	Unripe								
MAS-YOLO	97.5	95.3	92.4	89.0	96.4	82.3	91	2714936	12.8	5.9
**MIRB**	**97.5**	**95.8**	92.0	89.8	**96.7**	**82.8**	91	**2330168**	**6.3**	**5.1**
Conv2Former	97.2	94.8	91.1	89.3	96.0	80.8	90	2566712	11.4	5.5
DynamicConv	94.4	95.2	92.0	89.6	96.3	82.2	91	2471256	7.5	5.4

The results indicate that the Detect_MIRB head was the only one among the three lightweight designs to improve accuracy (mAP: 96.7%; mAP@0.5:0.95: 82.8%) while maintaining superior lightweighting characteristics. To accurately demonstrate the efficacy of this improvement, we quantified the Parameters, GFLOPs and time count layer-by-layer in the improved network (MASB-YOLO). These specific data are summarized in **Table 8**.

**Table 8:** Layered data for MAS-YOLO and MASB-YOLO networks. “/” denotes unchanged.

Layer	Time(ms)		FLOPs (G)		Parameters		Layer	Time(ms)		FLOPs(G)		Parameters		
Conv	1.56	1.56	0.1	/	464	/	A2C2f	1.56	/	0.31	/	24000	/	
Conv	0	0	0.24	/	4672	/	up	0	/	0	/	0	/	
C3k2	0	1.56	0.35	/	6640	/	Concat	0	/	0	/	0	/	
Conv	1.56	0	0.48	/	36992	/	A2C2f	1.56	/	1.04	/	19904	/	
C3k2	0	1.56	0.34	/	26080	/	Conv	0	/	0.48	/	36992	/	
Conv	0	/	0.47	/	147712	/	Concat	0	/	0	/	0	/	
A2C2f	6.25	/	0.57	/	174720	/	A2C2f	0.47	1.92	0.26	/	19904	/	
Conv	0	/	0.24	/	295424	/	Conv	1.56	0.2	0.12	/	36992	/	
A2C2f	4.69	/	0.55	/	677120	/	Concat	0	/	0	/	0	/	
ASAM	0	/	0	/	0	/	A2C2f	1.56	/	0.24	/	74624	/	
up	0	/	0	/	0	/	Conv	0	/	0.12	/	147712	/	
Concat	0	/	0	/	0	/	Concat	0	/	0	/	0	/	
A2C2f	1.56	/	0.28	/	86912	/	C3k2	1.56	/	0.30	/	378880	/	
up	0	/	0	/	0	/	Head	4.69	7.81	6.28	1.76	519192	134424	
Concat	0	/	0	/	0	/	Total	28.6	33.4	12.8	8.25	2714936	2330168	

The data presented above indicated that the MIRB head, by leveraging depthwise separable convolution, successfully reduced both the number of model Parameters (-16.5%) and GFLOPs (-50.8%). This very architectural choice. However, its highly sequential nature impaired parallel processing efficiency on hardware and increased memory access frequency. This limitation led to a slight increase in inference time. In this work, the MIRB structure is adopted to construct the detection head, replacing conventional standard convolution layers. This substitution substantially reduces both the number of parameters and computational overhead. While maintaining a lightweight architecture, the inverted residual structure enhances representational capacity and nonlinearity, enabling the detection head to more effectively capture subtle feature variations of fruits under low-contrast backgrounds.

### 3.5 Ablation experiment

To validate the contribution of each key component within the proposed method, a series of ablative studies were conducted. We term our proposed framework Collaborative Feature Refinement Detector (CFRDet). The name reflects two core design principles: (1) The four components are not independent; they jointly resolve the triple conflicts of near-color background, tiny objects, and lightweight requirement. (2) The network progressively refines feature representations—first by preserving weak gradients (Mish), then suppressing clutter (ASAM), recovering spatial details (small target layer), and finally compressing efficiently (MIRB). The performance of each variant was quantitatively evaluated using standard metrics under identical experimental settings. The specific data are shown in **Table 9**.

**Table 9:** Results of ablation experiments.

Module	Mish	ASAM	Small	MIRB	P (%)	R (%)	mAP (%)	mAP@0.5:0.95(%)	F1 (%)	Parameters	FLOPs (G)	Size (MB)
YOLOv12n	×	×	×	×	90.2	86.4	95.0	77.8	88	2520054	6	5.4
M-YOLO	√	×	×	×	90.5	88.3	95.4	79.2	89	2520054	6	5.4
M-A-YOLO	√	√	×	×	91.4	88.8	95.9	80.4	90	2550006	6.5	5.5
M-S-YOLO	√	×	√	×	91.1	89.3	96.0	80.8	90	2714936	12.8	5.9
M-B-YOLO	√	×	×	√	89.9	87.8	95.1	78.6	89	2229366	5.2	4.8
M-A-S-YOLO	√	√	√	×	92.4	89.0	96.4	82.3	91	2714936	12.8	5.9
M-S-B-YOLO	√	×	√	√	92.5	88.7	96.3	82.3	91	2330168	6.3	5.1
M-A-B-YOLO	√	√	×	√	92.1	88.5	96.2	81.1	90	2229366	5.2	4.8
MASB-YOLO (CFRDet)	√	√	√	√	92.0	89.8	96.7	82.8	91	2330168	5.6	5.1

**Figure 10:**
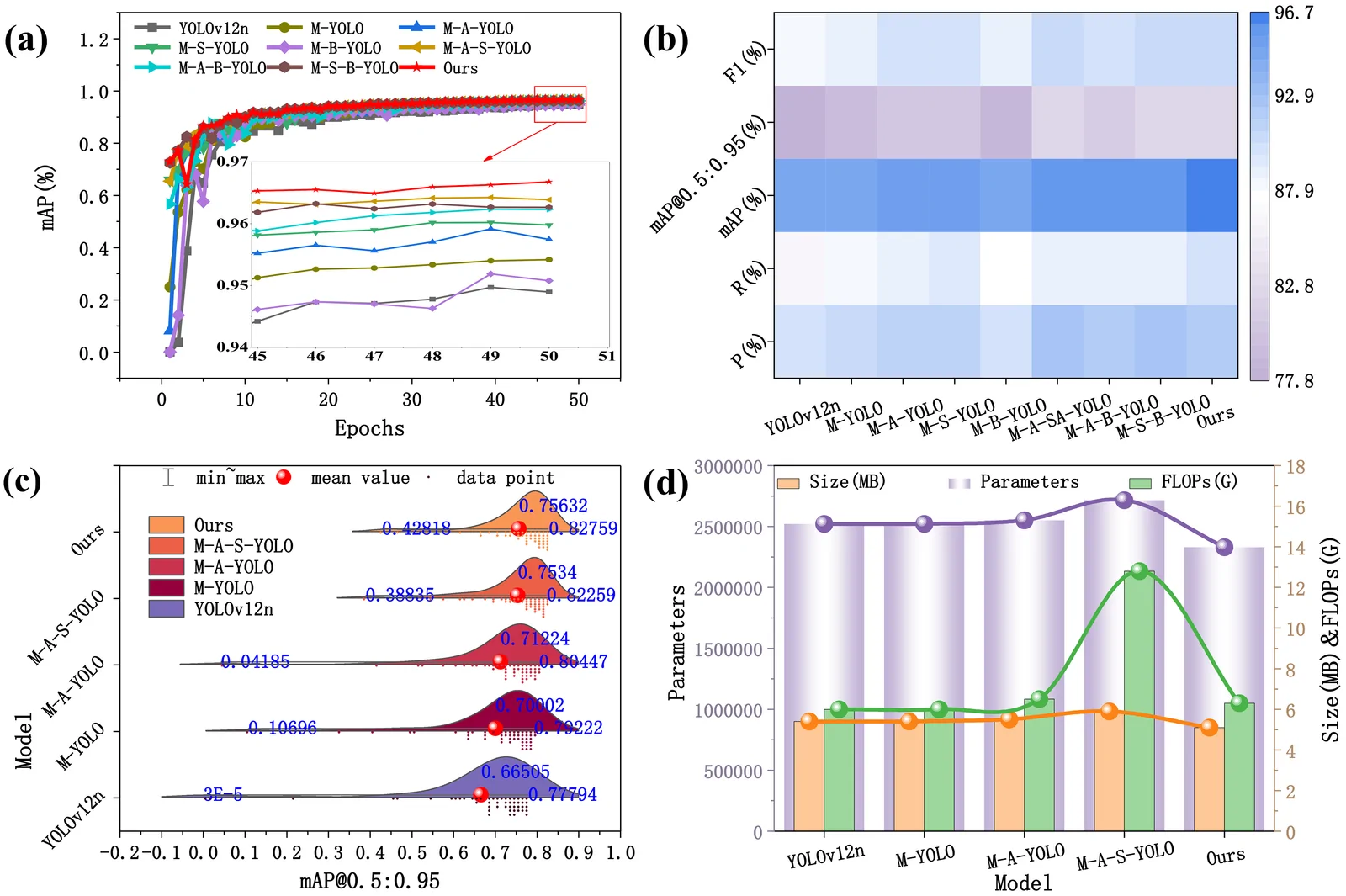
Visualization of ablation experiment results. **(a)** Comparison of mAP of each scheme. **(b)** Comparison of all precision indicators of each scheme. **(c)** Comparison of mAP0.5:0.95 for sequential ablation schemes. **(d)** Comparison of complexity of sequential ablation schemes.

The experimental results demonstrated the effectiveness of our stepwise improvement strategy. Initially, the Mish activation function was introduced to establish a stronger baseline, which elevated the mAP and mAP@0.5:0.95 to 95.4% and 79.2%, respectively. The Mish activation function, with its continuous, smooth, and non-monotonic properties, facilitates improved gradient flow and enriches feature representation. This enables the network to learn more discriminative fine-grained features while enhancing sensitivity to small-target characteristics and strengthening the model baseline. Subsequently, after comparing the effectiveness and training cost of three candidate enhancements, the parameter-free ASAM attention module was incorporated to improve feature representation quality. Employing the intrinsic energy of feature maps to generate 3D attention weights, the ASAM module (which introduces no parameters) amplifies local feature disparities and suppresses homogeneous background regions. This mitigation of feature submersion, coupled with its reinforcement of fine-grained discriminative and contextual feature perception, allows the model to effectively differentiate between globally analogous but locally distinct regions. Then, the addition of a dedicated small-object detection layer further increased mAP and F1-score by 1.4% and 3.0%, respectively. By preserving high-resolution spatial details, the small-target detection layer enhances architectural suitability for small objects and supports the differentiation of color-similar regions. Finally, to achieve a balance between detection accuracy and model efficiency, the MIRB-based detection head was adopted. Compared to the original baseline, the final model achieved increases of 1.7% in mAP and 5% in mAP@0.5:0.95, while reducing parameter count and model size to 92.5% and 94.4% of the original, respectively. The effectiveness of each improvement scheme is summarized in **Figure 10**.

In the ablation study, the CFRDet achieved the highest mAP compared to the baseline YOLOv12n and the other 8 improved variants (the red solid curve in **Figure 10a**). As anticipated, the introduction of M-B-YOLO, while achieving model lightweighting, resulted in a reduction in detection accuracy. Meanwhile, all other accuracy metrics also reached their maximum values, which is corroborated by the heatmap presented in **Figure 10b**. Furthermore, sequential ablation experiments conducted in this study demonstrated that the CFRDet network achieved the highest mAP@0.5:0.95 among the 5 ablation configurations, as depicted in the violin plot in **Figure 10c**. It can also be observed from the complexity trends in **Figure 10d** that after incorporating the MIRB detection head, the model parameters, GFLOPs, and model size were all reduced.

#### 3.5.1 Ablation on ASAM and MIRB

To avoid simple superposition of existing technologies, we must validate whether the ASAM and MIRB modules address specific sub-problems in small target detection under near-color background conditions regarding high accuracy and lightweight performance, while ensuring their combination generates genuine synergistic effects rather than redundancy. **Table 10** reports the results.

**Table 10:** Ablation experiment results of ASAM and MIRB.

Module	ASAM	MIRB	mAP (%)	mAP@0.5:0.95(%)	F1 (%)	Parameters	FLOPs (G)	Size (MB)
Baseline	×	×	95.4	79.2	89	2520054	6	5.4
+ASAM	√	×	95.9 (+0.52%)	80.4 (+1.52%)	90 (+1.12%)	2550006 (+1.19%)	6.5 (+8.33%)	5.5 (+1.85%)
+MIRB	×	√	95.1 (-0.31%)	78.6 (-0.76%)	89 (+0)	2229366 (-11.53%)	5.2 (-13.33%)	4.8 (-11.11%)
+ASAM&MIRB	√	√	96.2 (+0.84%)	81.1 (+2.4%)	90 (+1.12%)	2229366 (-11.53%)	5.2 (-13.33%)	4.8 (-11.11%)

**Table 10** presents the contribution of each module. The baseline already achieves strong performance (95.4% mAP). Adding ASAM alone improves mAP by 0.5% and mAP@0.5:0.95 by 1.52%, with a slight increase in FLOPs (+8.33%). This confirms ASAM’s effectiveness in suppressing color-similar background (leaves and unripe fruits) while preserving tiny jujube responses. Adding MIRB alone reduces parameters by 11.53% and FLOPs by 13.33%, but causes a small drop in mAP (-0.3%) and mAP@0.5:0.95 (-0.76%), indicating that aggressive lightweighting harms the detection of tiny, low-contrast targets. When combined, ASAM and MIRB achieve the best mAP (96.2%) and mAP@0.5:0.95 (81.1%) while maintaining the full lightweight benefit of MIRB (4.8 MB, 5.2 GFLOPs). This synergy arises because ASAM’s parameter-free attention compensates for the feature degradation caused by MIRB’s depthwise convolutions, yielding a Pareto improvement over both individual additions.

#### 3.5.2 Visualization effect validation

**Figure 11:**
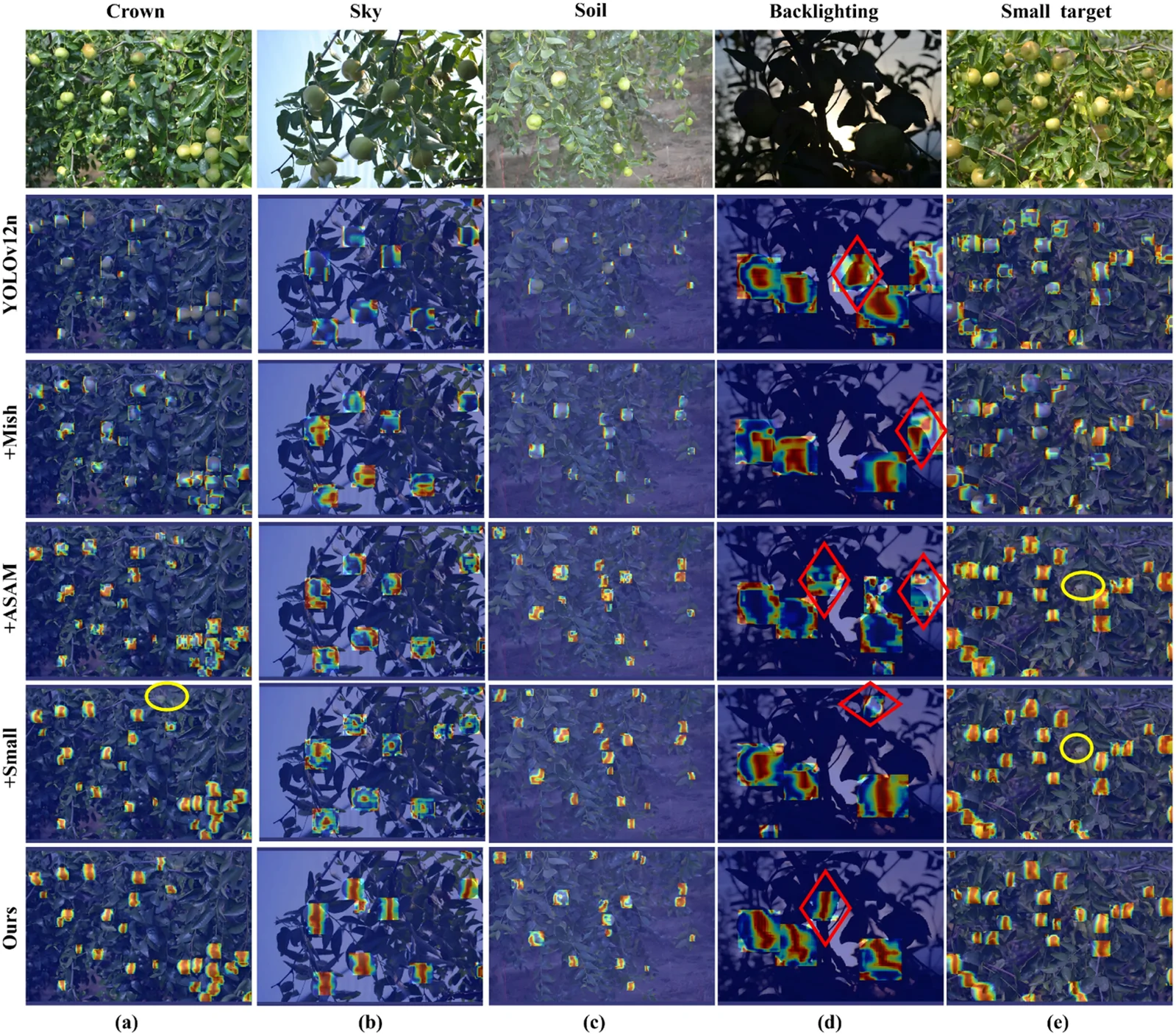
Visual comparison of activation heatmaps. Yellow circles: missed detections. Red diamonds: false detection.

To verify that our architectural enhancements indeed guide the model to focus more accurately on targets without being distracted by similarly colored backgrounds, we conducted a visual comparison of activation heatmaps between the baseline and our proposed method. As illustrated in **Figure 11**, the baseline model produced a diffuse activation pattern that spread beyond the actual boundaries of the target fruit (red diamonds). In contrast, our model generated a concentrated and well-localized heatmap, which aligns closely with the true object contours (Line 6). This result demonstrates the improved capacity of our approach to precisely identify and localize regions of interest.

When the target color resembled the background, baseline activations remained ambiguous and poorly localized (row 2). The introduced ASAM module effectively suppressed background noise and confined the network’s attention to the discriminative features of the target. This refined focus, evident in the sharper activation profiles (**Figure 11**, rows 3a, 3d, 3e), directly reduced false positives caused by near-color background. Furthermore, in SOD task (**Figure 11e**), the baseline detector exhibited weak and sporadic attention, resulting in missed detections. By contrast, our model (enhanced by the fine-grained feature amplification in MIRB) produced strong, distinct activations for each small target, consistent with the higher recall reported in **Table 9**.

We conducted a heatmap analysis to compare the baseline network and the four incremental modules: +Mish, +ASAM, +Small Layer, and +MIRB. The baseline missed some targets (rows 2a, 2c, 2e) and also produced false positives (rows 2d, 2e). Adding Mish improved the model’s attention focus on the jujubes. However, the ASAM module became less effective under near color backgrounds, as shown in row 4d where attention shifted to the background. One potential reason was that, under extreme color similarity, the feature vectors of the fruits and the background almost overlapped in the high−dimensional space, so ASAM incorrectly suppressed them together. Finally, the full model still suffered from a few missed detections (row 6c) and false positives (row 6d). This suggests that while the collaborative design reduces errors, challenging conditions such as extreme color similarity and dense clusters remain difficult for our network.

#### 3.5.3 Statistical significance analysis

To ensure that the improvement effect was not caused by random factors, we conducted 5 independent runs of the full model on the flat jujube dataset using KFold cross-validation, and the performance was reported as mean ± standard deviation. The results are summarized in **Table 11**.

**Table 11:** Validation results of the KFold cross-validation.

KFold cross-validation	P(%)	R(%)	F1(%)	AP(%)		mAP (%)	mAP@.5:.95 (%)
				Ripe	Unripe		
Fold 1	92.5	89.3	91	97.4	94.5	96.4	82.4
Fold 2	92.7	89.1	91	97.5	94.5	96.5	82.5
Fold 3	92.0	89.0	91	97.4	93.8	96.7	82.8
Fold 4	91.2	89.4	91	97.1	94.1	96.9	83.2
Fold 5	93.0	89.2	91	97.7	94.6	96.7	83.1
Mean ± Std	92.0 ± 1.0	89.0 ± 0.4	91 ± 0.0	97.4 ± 0.3	94.1 ± 0.5	96.7 ± 0.3	82.8 ± 0.4

The results presented in the table above indicated that the CFRDet achieved an mAP of 96.7% (± 0.3) across 5 rounds of KFold cross-validation. The small standard deviation reflected stable performance, with no notable fluctuation arising from the particular split between training and validation sets. These results demonstrate that the advantages of CFRDet are robust and not incidental.

### 3.6 Comparative experiment on mainstream networks

To ensure a fair comparison, we used the same hyperparameters for all baseline methods. We trained and tested every model on the same dataset under identical settings. No method received any additional tuning or preferential treatment. This guaranteed that the performance differences came from the network architectures themselves, not from the training settings.

A set of 12 mainstream models was selected for comparison to rigorously evaluate the competitive performance and effectiveness of the proposed CFRDet. These models included: the two-stage network Faster R-CNN, the one-stage networks RT-DETR-R18 and SSD, a peer nano-scale models, a mobile variants with the backbone replaced by the MobileOne re-parameterization module, a pruned variants (at 30% sparsity), and small-scale models of corresponding versions. In addition, a comparative analysis of the latest state-of-the-art (SOTA) lightweight agricultural object detectors, including YOLOv5n, YOLOv8n, YOLOv12n, SOD-YOLO and v12n-pruned was also presented. The corresponding results are summarized in **Table 12**.

**Table 12:** Comprehensive comparison between improved networks and mainstream networks.

Scheme	AP(%)		P (%)	R (%)	mAP(%)	mAP@0.5:0.95(%)	F1 (%)	Parameters	FLOPs(G)	Size (MB)
	Ripe	Unripe								
Faster RCNN	96.3	92.4	90.6	85.9	94.4	72.7	88	41685135	207.0	159.7
SSD	96.4	92.2	89.2	86.4	94.3	77.5	88	27135416	35.2	91.1
DETR-R18	96.7	92.4	89.4	87.4	94.5	77.8	88	20321685	58.6	38.6
YOLOv5n	96.3	91.9	91.1	84.8	94.1	72.1	88	1766623	4.2	3.7
SOD-YOLO	96.1	91.0	89.3	85.3	93.5	76.3	87	1571292	4.1	3.3
YOLOv8n	97.0	93.3	89.7	88.5	95.1	78.9	89	3011386	8.2	6.2
YOLO-DLHS-P	96.3	91.9	89.0	85.7	84.1	77.2	87	2009510	5.4	4.2
YOLOv10n	96.7	93.3	90.5	88.0	95.0	78.8	89	2707647	8.4	5.7
YOLOv11n	96.3	92.1	89.4	85.9	94.2	77.3	88	2590865	6.4	5.4
YOLOv12n	96.7	93.3	90.2	86.4	95.0	77.8	88	2520054	6.0	5.4
v12n-pruned	94.9	88.4	85.5	83.9	91.6	71.6	85	1274902	4.1	2.8
v12n-PConv	96.1	91.0	89.3	85.3	93.5	76.3	87	1571292	4.1	3.3
CFRDet	97.5	95.8	92.0	89.8	96.7	82.8	91	2330168	5.6	5.1
YOLOv12s	97.4	95.3	91.9	90.0	96.3	82.4	91	9097546	19.6	18.6
YOLOv13n	96.5	93.0	90.1	86.4	94.7	77.5	88	2460301	6.4	5.4

**Figure 12:**
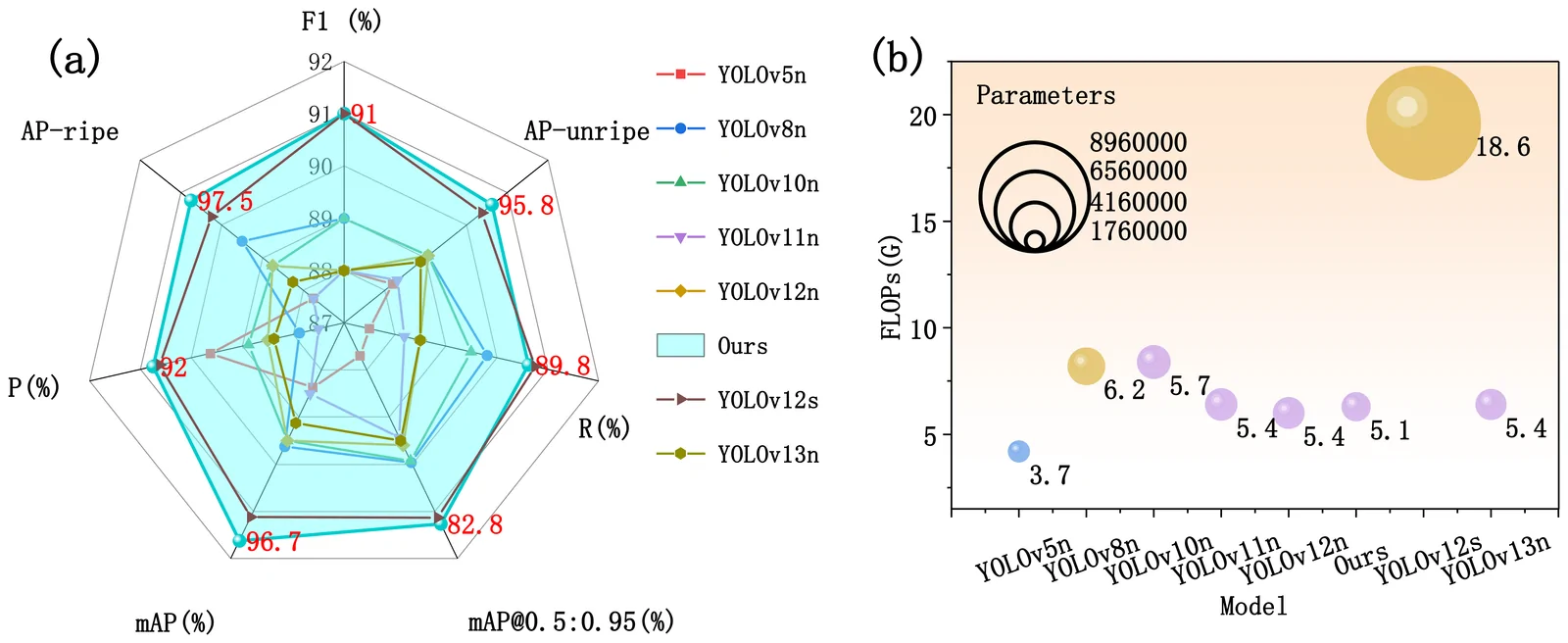
Experimental results compared with mainstream networks. **(a)** Comparison of accuracy indicators of each network. **(b)** Comparison of complexity indicators of each network.

The data in **Table 12** indicate that while SOD-YOLO, YOLOv12n-pruned yielded the best lightweighting results, it also had the lowest precision (mAP: 94.1%, 91.6%). In contrast, other nano-scale models including YOLOv8n, v10n, and v12n achieved mAP above 95%, among which YOLOv12n demonstrated superior complexity characteristics. On this basis, the proposed CFRDet attained the maximum mAP and mAP@0.5:0.95 values of 96.7% and 82.8%, outperforming even the YOLOv12s in accuracy, yet with only 25.6% of the parameters, 42.3% of the GFLOPs, and 27.4% of the model size. **Figure 12** summarizes the trend changes of the YOLO series from the table above.

As illustrated by the cyan area in the radar chart (**Figure 12a**), CFRDet led all other leading networks across every precision metric. Regarding computational complexity, the bubble chart in **Figure 12b** shows that parameters and GFLOPs exhibited minor variations among the models, excluding the extremes represented by YOLOv12s (largest) and YOLOv5n (smallest). Furthermore, CFRDet achieved the smallest model size alongside its superior accuracy. In summary, the proposed CFRDet demonstrated the most comprehensive detection performance.

### 3.7 Validation of model detection effect and generalization ability

**Figure 13:**
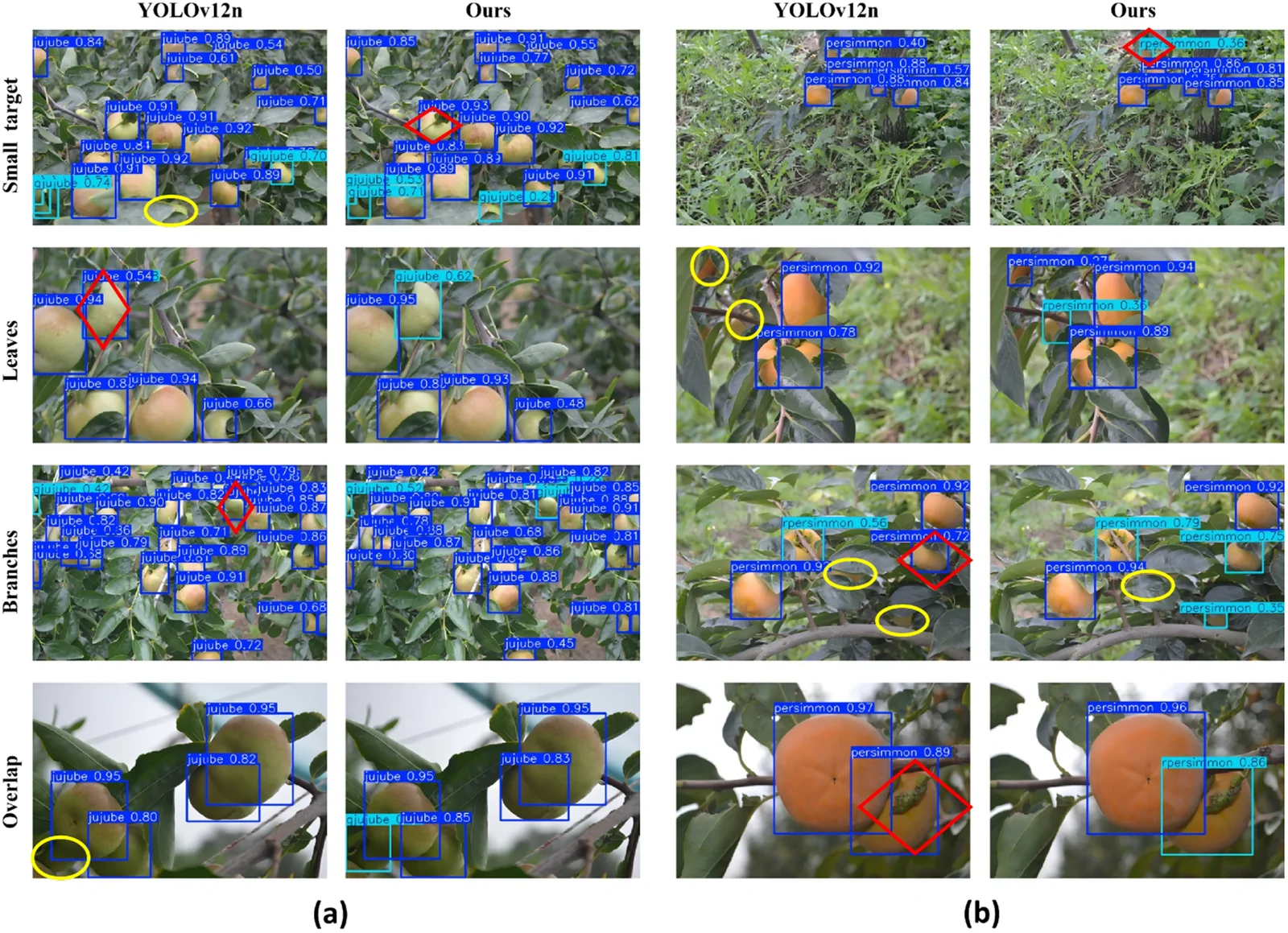
Model detection effect. **(a)** Oblate jujube. **(b)** Sweet persimmon. Yellow circles: missed detections. Red diamonds: false detections.

The model’s effectiveness was validated using field-acquired jujube images, while its generalization capability was tested on persimmons, a similarly colored fruit. Sample detection results are illustrated in **Figure 13**.

As evidenced by the graphical results, YOLOv12n exhibited generally weaker detection accuracy relative to the improved CFRDet framework, along with instances of missed and false detections. By incorporating the Mish activation function, CFRDet enhanced its capability to capture fine-grained features of fruits with similar coloration. The addition of a dedicated small-object detection layer and the ASAM attention mechanism further improved the recognition performance for small and near-color fruits, as indicated by the cyan labels in the figures.

Nevertheless, the CFRDet framework was not immune to detection errors, and was still observed to produce duplicate or false detections, particularly in densely clustered environments, as shown in the second and fourth columns of **Figure 13**. These challenges stemmed from the inherently complex and unstructured nature of agricultural environments. Severe occlusion often led to incomplete feature extraction or even misclassification, while the presence of small-sized and similarly colored targets further increased the difficulty of accurate detection.

## 4 Conclusion

This study has presented CFRDet, an innovative architecture specifically designed for detecting small objects with color-similar backgrounds in complex agricultural environments. First, Mish is adopted as the core activation to maintain a gentle gradient flow for weak features, preventing them from being zeroed out (unlike ReLU). Second, ASAM leverages these retained weak responses to compute 3D attention weights without introducing any parameter overhead, effectively suppressing the confusing background while enhancing the target. Third, a tiny-object detection layer is appended to recover spatial details lost in downsampling, but this would normally increase parameters and FLOPs dramatically. Fourth, detection heads with MIRB and depthwise convolutions are specifically designed to process this high-resolution feature map at a nearly linear computational cost. In this way, each component compensates for the side effects of the others, forming a virtuous cycle: MIRB makes the tiny-object layer affordable, ASAM cleans its noisy features, and Mish ensures that the cleaned features survive through the deep network.

Our findings indicate that CFRDet not only surpassed all compared nano-scale models in terms of mAP and mAP@0.5:0.95 but also exhibited superior or comparable accuracy to small-scale models (96.7% and 82.8%), while maintaining a significantly reduced parameters (-74.4%) and computational load (-67.9%). Furthermore, a SOTA comparison of the latest lightweight agricultural object detectors (SOD-YOLO, YOLO-DLHS-P, YOLOv12n-PCONV, etc.) is included in this work. This breakthrough suggests that careful architectural redesign and detailed model optimization, rather than simply scaling up the model, is a viable path toward developing highly efficient vision systems for edge-computing platforms in precision agriculture. This research is dedicated to advancing the application of AI in agricultural automation, providing more adaptive and intelligent references for computer vision in the task of field-based agricultural product detection.

Despite its promising results, this work has certain limitations. (1) We collected our dataset from a single orchard under limited lighting conditions. As a result, we do not know if the model works well for other jujube types or in bad weather. To fix this, future work will collect more data from different orchards and varieties. We will also use domain adaptation methods. (2) **Figure 11d** illustrates a challenging backlit scenario. While all compared models exhibited limited performance, our method maintained measurable activation on the visible portions of the target (Line 6d), whereas the baseline model’s attention shifted entirely to the overexposed foreground. This residual activation capacity explains the model’s tendency to yield partially valid detections under such conditions, although performance degradation under extreme occlusion remains. Future work could be directed toward integrating non-visible spectral data (e.g., multispectral or thermal imaging) with semantic or geometric context to mitigate false and missed detections in backlit or occluded scenes. (3) We set the resolution of the tiny object detection layer to 160 by 160 based on experience. We did not try to find the best balance between accuracy and speed. Future research will look into adaptive resolution scaling and model compression like pruning or quantization. This will help the model run on very lightweight edge devices. (4) Our work only evaluates detection performance alone. We have not combined it with a full harvesting robot system that does 3D localization or grasp planning. Future work will put CFRDet into a real robot prototype and test it in the field. Addressing these limitations will make our collaborative feature refinement approach more useful in practice.

## Data Availability

The raw data supporting the conclusions of this article will be made available by the authors, without undue reservation.
